# The Abuse and Use of Bromides

**Published:** 1877-06

**Authors:** 


### MISCELLANEOUS.

### New York Medical Journal Association.

Stated Meeting April 20, 1877. Dr. CHARLES M. ALLIN, President, in the Chair.

### THE ABUSE AND USE OF BROMIDES.

Dr. E. C. Seguin, in an interesting and valuable paper, discussed the above subject under two heads: 1. Bromism, or intoxication by the bromides. 2. A statement of his own method of using the bromide salts in the treatment of epilepsy and other neuroses. Since the bromides had been brought into use in the treatment of neuroses, especially hysteria and epileysy, by Sir Charles Locock (1857), and systematized by Brown-Séquard, they had been employed in the treatment of an almost endless list of diseases and symptoms. The general use of the various bromides was largely empirical, the medicine being prescribed because of its quieting effect, and regardless of its physiological action.

Since 1867 experimental physiologists had been making researches regarding the effect of bromides upon the healthy organism, and the most important conclusions reached were two in number. By some the bromides were believed to act by causing contraction of the arterioles and consequent diminution in the amount of blood in the nervous centres; while others claimed that they affected the nervous tissues directly. There was a general agreement, however, that the physiological result of the action of the bromides was lessened irritability of the nervous centres, especially in the motor tract.

Dr. Seguin believed that the bromides acted mainly by affecting the anatomical elements (ganglion cells chiefly) of the central nervous system. His belief was based upon physiological experiments in animals, clinical observations in man, and largely by the phenomena of bromism, which could hardly be explained by the vascular theory of the action of the bromides.

It was believed to be chiefly in consequence of the empirical notion that the bromides were indicated whenever there was excitement, aided probably by the extreme application of certain theoretical views regarding the physiological importance of changes in the amount of blood in the brain and spinal cord, that there had been, and still continued, an abuse or over-use of the various bromides. Cases were not infrequent in which a condition of impaired nutrition and nervous atony had been brought about and continued for months or years by the use of these medicines.

At this point Dr. Seguin divided his remarks into three sections: 1, concerning the general description of mild and of severe bromism; 2, respecting the complication which bromism might cause in diagnosis; and 3, with reference to the legal aspects of bromism.

### VARYING DEGREES OF BROMISM.

In a number of cases Dr. Seguin had observed the following symptoms superadded to the legitimate symptoms of the disease: general debility, with weak pulse and coldness of the extremities; tendency to stupor; slight difficulty in speaking; the bromic breath, and acne. Those patients, were weak, anæmic individuals, who had been taking the bromides for the relief of certain head symptoms, gratuitously supposed to be due to cerebral congestion. In some cases moderate doses of the drug had been taken for long periods of time, with frequent temporary relief to certain symptoms. All the time, however, the general condition of the patient had been kept below par, in spite of tonics and selected food. The same mild bromism had been noticed in some cases of hysteria and hystero-epilepsy, without any actual improvement. Injurious effects had been seen from the prolonged use of the bromides in the treatment of melancholia, a disease in which cerebral nutrition was quite surely lowered and perverted. Reference was made to a large class of patients who, without definite disease, suffered from nervousness, inability to sleep, queer sensations about the head, and who were inclined to constantly over-estimate their symptoms. To such patients the physician or druggist was very apt to say, "Take a dose of the bromides." It might be said that the administration of the bromides in such manner did not produce positive ill effects; but to that assertion Dr. Seguin replied by saying, *first*, that from what was known of the physiological effects of the bromides, such dosing must produce a general lowering of vitality which few patients could tolerate; and, *second*, that on principles, physicians were in duty bound to give no superfluous or non-indicated drug to their patients.

Allusion was then made to the more severe forms of bromism, in which the condition might obtain the dignity of a definite morbid state, have a clear symptomatology, a well-known course, and, as the doctor was disposed to believe, a central lesion. The symptoms of the severe forms of bromism might be so aggravated as to similate dementia, mania, or general paralysis of the insane, and even death might ensue from debility.

Dr. Seguin drew special attention to the resemblance between bromism and general paralysis of the insane. In both there was tremor of the facial and lingual muscles, producing a peculiar vibratory speech; in both there was an uncertainty in the performance of certain movements, as walking or using the hands for fine work; in both there was failure of intellectual force and of memory. Even somewhat exalted notions, though rarely, might be present in bromism. In general paralysis there were other important symptoms, such as contraction and irregularity of the pupil, sexual excitement, peculiar epileptiform seizures, remarkable remissions in the symptoms, and often good physical health, with tense arteries; all those symptoms being absent in bromic intoxication.

Severe bromism fortunately was rarely seen except in the early stage of the treatment of obstinate epilepsy. Bromism had been proposed as a cure for the opium habit, and the paper written by Dr. Schweig, of New York, upon that subject furnished a valuable study of the severe effects of the bromides.

### BROMISM AS A COMPLICATION IN DIAGNOSIS.

Under that head reference was made by Voisin. The patient had been under treatment for epilepsy, and, as his physician thought, becoming insane, was sent to Paris. He had been taking during some months bromide of potassium in doses of 90 to 120 grains. The patient was in a state of violent mania, and was removed to the asylum, where his case was looked upon by the officers of the institution as one of general paralysis of the insane. After the cessation of the bromides and the adoption of proper treatment, the man, at the end of thirteen days, was sent to his home in the country quite well.

Dr. Sequin then gave the history of a case which came under his observation, and in which the addition of bromism to the other symptoms had led to the diagnosis of cerebral lesion of the gravest kind, when really only the basal dura mater was involved. The bromides were withheld and the iodide of potassium substituted, to give the patient the benefit of a doubt regarding the presence of a specific element. The symptoms of cerebral leision passed away in a few days, and the local symptoms gradually disappeared, except the atrophy of the optic nerve.

### THE MEDICO-LEGAL ASPECT OF BROMISM.

Bromism, although it has not been brought into the courts, might at some time appear under several circumstances.

*First*, with reference to the responsibility of the physician administering a medicine which produced such mental and physical debility as to expose the patient to various mishaps. For instance, a patient suffering from acute bromism had fallen asleep in a railway station and was robbed of four hundred dollars.

*Second*, with reference to the responsibility of the patient for committing criminal acts while suffering from bromism.

It was believed to be perfectly possible for such patients to take articles not paid for, through defective memory; to be mistaken regarding the identity of persons, and thus be led to be abusive, etc.

*Third*, with reference to the legal capacity of brominized persons.

In some cases of bromism the stupor, loss of memory, and aphasiform difficulty were so great that the patient was as truly *non compos mentis* as if he had a natural secondary dementia.

In certain cases it would be very difficult to reach a correct decision, because the judgment and general intellection were remarkably well preserved behind a display of superficial symptoms; and it also might be difficult to determine how much of the mental impairment depended upon the patient's antecedents, and how much upon the disease for which the medicine was taken.

*Fourth*, with reference to the production of death through bromism. There was a possibility that the procedure might be repeated with criminal intentions; perhaps for the purpose of getting rid of an incurable invalid.

### METHOD OF USING THE BROMINE SALTS IN THE TREATMENT OF EPILEPSY AND OTHER NEUROSES.

1. The prolonged use of bromides was contraindicated by congenital feebleness.
2. The bromides were well borne by persons of fairly full habit and good nervous power.
3. The bromides were indicated in cases of abnormally great irritability of the nervous system in its motor (muscular and vasomotor, and ideational tracts.
4. The contraindications above named were to be much less regarded in the management of that formidable neurosis, epilepsy.
5. Epilepsy were regarded as the only diisease which justified the deliberate production of a degree of bromism for its cure.

Dr. Seguin's method of prescribing the bromides in the treatment of a case of "idiopathic" epilepsy was the following:

Two solutions were employed.

℞ Potassii bromidi. . . . ℥ i.
Ammon. bromidi. . . . . ℥ ss.
Aquæ font. . . . . ℥ vij.
M.

S. To be given by the teaspoonful.

And

℞ Sodii bromidi . . . . ℥ i.
Ammon. bromidi . . . ℥ ss.
Aquæ font. . . . . . ℥ vij.
M.

S. To be given by the teaspoonful.

The quantity administered was, as a rule, so divided as to give by far the largest dose in the evening. The bromide was cautiously increased, still keeping the nocturnal dose the largest, until slight bromism was produced. It was usually necessary to maintain slight bromism for months, but just as little was to be given as would prevent the attacks. The precise quantity required must be studied in each case. Children tolerated the bromides, as well as the iodides, in relatively large doses. It was regarded as important to thoroughly dilute the bromides in order to facilitate their absorption—the dose to be taken in a wineglassful or half a tumblerful of water. Under no circumstances should the bromides be discontinued; they might be *diminished*, but not *stopped* until the word *cure* could be pronounced. They should be continued at least three years after the last attack. The adjunct treatment consisted in the use of measures to prevent the acne to a certain extent, such as the occasional use of arsenic, sulphur ointments, mercurial plaster, and alkaline lotions; to correct the general debility or slight paresis, by the use of strychnia, nux vomica, oxide of zinc, and quinia; to relieve the dizziness by the inhalation of nitrite of amyl, by stimulants, and by quinia; regulating the patient's diet and hygiene, and the use of cream, codliver oil, iron, quinia, phosphorus, strychnia, with nitro-muriatic acid, wine, deer, or whiskey. In certain cases such medicines as acted more directly upon the morbid state of the nervous centres were associated with the bromides, and the favorite among those was belladonna. In the treatment of cases of epilepsy in which a definite causative lesion could be made out, the bromides were used simply to combat the habit.

In the treatment of other neuroses, Dr. Seguin had used the bromides sparingly, and never continuously. Bromism should not be produced in the treatment of *hysteria*. *Delirium tremens* might probably be shortened by the free use of the bromides.

Many cases of *insommia*, treated upon the purely hypothetical indication of causing anæmia of the brain, might be much more quickly relieved by chloral, or by a glass of ale, or by correcting indigestion, than by the use of the bromides. Sleep was believed to be due partly to the general waste of tissues and the accumulation in the blood of the products of retrograde metamorphosis, and partly to the exhaustion of the cerebral tissue itself. The anæmia observed in the brain during sleep was regarded as a consequent phenomenon in obedience to the general law that a tissue repose in contained less blood than one in action.

In the treatment of *insanity*, the use of the bromides was recommended only to meet such indications as a tendency to epileptiform attacks, or abnormal sexual excitement, or great nervousness not caused by delusions. Favorable reference was made to the use of bromide of potassium before administering ether or opium with the view of preventing nausea and vomiting. Special attention was called to the use of bromide of ammonium in the treatment of *hay-asthma*—used as a gargle and to wash out the nasal passages several times a day with a weak solution of the same salt. The gargle was of the strength of ʒi or ʒij. to the ℥i. of water; the solution for the nares from 10 to 30 grains to the ℥i of water.—*Medical Record.*

